# miRNA-133b targets FGFR1 and presents multiple tumor suppressor activities in osteosarcoma

**DOI:** 10.1186/s12935-018-0696-7

**Published:** 2018-12-18

**Authors:** Gan Gao, Zhen Tian, Huan-Ye Zhu, Xun-Yan Ouyang

**Affiliations:** Department of Orthopedics, Guizhou Provincial People’s Hospital, No. 83, East Zhongshan Road, Guiyang, 550002 Guizhou People’s Republic of China

**Keywords:** Osteosarcoma, miR-133b, Fibroblast growth factor receptor 1, Tumor suppressor, Ras/MAPK signaling, PI3K/Akt signaling

## Abstract

**Background:**

Osteosarcoma (OS) is the most common bone malignancy prevalent in children and young adults. MicroRNA-133b (miR-133b), through directly targeting the fibroblast growth factor receptor 1 (FGFR1), is increasingly recognized as a tumor suppressor in different types of cancers. However, little is known on the biological and functional significance of miR-133b/FGFR1 regulation in osteosarcoma.

**Methods:**

The expressions of miR-133b and FGFR1 were examined by RT-qPCR and compared between 30 paired normal bone tissues and OS tissues, and also between normal osteoblasts and three OS cells lines, MG-63, U2OS, and SAOS-2. Using U2OS and MG-63 as the model system, the functional significance of miR-133b and FGFR1 was assessed on cell viability, proliferation, apoptosis, migration/invasion, and epithelial–mesenchymal transition (EMT) by overexpressing miR-133b and down-regulating FGFR1 expression, respectively. Furthermore, the signaling cascades controlled by miR-133b/FGFR1 were examined.

**Results:**

miR-133b was significantly down-regulated while FGFR1 robustly up-regulated in OS tissues and OS cell lines, when compared to normal bone tissues and normal osteoblasts, respectively. Low miR-133b expression and high FGFR1 expression were associated with location of the malignant lesion, advanced clinical stage, and distant metastasis. FGFR1 was a direct target of miR-133b. Overexpressing miRNA-133b or knocking down FGFR1 significantly reduced the viability, proliferation, migration/invasion, and EMT, but promoted apoptosis of both MG-63 and U2OS cells. Both the Ras/MAPK and PI3K/Akt intracellular signaling cascades were inhibited in response to overexpressing miRNA-133b or knocking down FGFR1 in OS cells.

**Conclusion:**

miR-133b, by targeting FGFR1, presents a plethora of tumor suppressor activities in OS cells. Boosting miR-133b expression or reducing FGFR1 expression may benefit OS therapy.

## Introduction

Osteosarcoma (OS) is the most common and a highly aggressive bone malignancy primarily developing in teenagers and young adults [1]. OS arises from transformed cells presenting osteoblastic differentiation and producing malignant osteoid [2]. Although surgical resection combined with neoadjuvant chemotherapy have reduced the mortality of OS patients, leading to the 5-year survival of approximately 65–70% for those without metastasis, OS patients with metastasis still suffer a dismal prognosis and the 5-year survival rate of only 10–20% [3, 4]. Therefore, it is critical to identify novel diagnostic biomarkers and/or therapeutic targets to achieve early diagnosis and effective treatment of this disease, with specific focus on targeting the metastasis of OS.

MicroRNAs (miRNAs) are short non-coding RNAs that bind to the 3′-untranslated region (3′-UTR) of a target mRNA, thus degrade the mRNA target, and/or induce translational silencing [5]. Through post-transcriptional and/or translational regulation on target genes, miRNAs critically regulate a plethora of physiological and pathological processes. In cancers, miRNAs act, either positively or negatively, on all the hallmarks of cancers, namely “sustaining proliferative signaling, evading growth suppression, resisting cell death, enabling replicative immortality, inducing angiogenesis, and activating invasion and metastasis” [6]. The abnormal expressions of certain miRNAs are closely associated with the diagnosis, the prognosis, and the treatment response of different types of human cancers and thus miRNAs become important diagnostic and prognostic biomarkers as well as therapeutic targets for cancers [7]. Of the pool of cancer-related miRNAs, miR-133b is a canonical muscle-specific miRNA that is physiologically critical for the development of mammalian skeletal and cardiac muscles [8]. Recent studies revealed the tumor suppressor activities of miR-133b in different human cancers [9]. In OS, miR-133b expression was significantly reduced and re-introduction of miR-133b in osteosarcoma cells inhibited cell proliferation, induced apoptosis, and suppressed migration/invasion [10]. However, the molecular mechanisms underlying the anti-cancer activities of miR-133b in OS are not well understood.

The fibroblast growth factor receptor 1 (FGFR1), together with FGFR2, FGFR3, and FGFR4, constitute the FGFR family of receptor tyrosine kinases and convey the signals of different fibroblast growth factors (FGFs) intracellularly [11]. By activating four major signaling cascades, Ras/mitogen-activated protein kinase (MAPK), phosphatidylinositide 3-kinases (PI3K)/Akt, Phospholipase C gamma (PLCγ), and the signal transducers and activators of transcription (STAT), FGFRs essentially control cell growth, proliferation, survival, differentiation, and angiogenesis [11, 12]. Genetic variations, including gene amplification and chromosomal translocation, increase FGFR1 expression and make it an ideal therapeutic target in several human cancers [13]. In gastric cancer, the expression of miR-133b was inversely correlated with that of FGFR1, bioinformatic analysis revealed FGFR1 was a direct of miR-133b, and by targeting FGFR1, miR-133b inhibited the growth of gastric cancer cells [14]. In contrast, little is known on the biological and clinical significance of miR-133b/FGFR1 axis in OS.

In this study, we compared the expressions of miR-133b and FGFR1 in human OS tissues vs matching normal bone tissues, and also in OS cell lines vs normal osteoblasts. We showed that FGFR1 was a direct target gene inhibited by miR-133b in OS cells and their expressions were significantly correlated with location of the malignant lesion, higher staging or the presence of metastasis in OS patients. We analyzed the significance of overexpressing miR-133b or down-regulating FGFR1 in regulating the viability, proliferation, apoptosis, migration/invasion, and epithelial–mesenchymal transition (EMT) of OS cells. Furthermore, we explored the signaling cascades altered by miR-133b/FGFR1 axis in OS cells, specifically PI3K/Akt and Ras/Raf/Erk pathways. By targeting FGFR1, miR-133b became a pleiotropic tumor suppressor miRNA, inhibiting the tumorigenic as well as metastatic behaviors of OS cells. Therefore, boosting the expression of miR-133b or reducing that of FGFR1 may prove a promising therapy for OS.

## Materials and methods

### Human samples

This study was approved by the Ethics Committee of Guizhou Provincial People’s Hospital (Guiyang, Guizhou, China) and written consent was obtained from all participants. A cohort of 30 OS patients were recruited into this study. The OS tissues and paired normal bone tissues were acquired during surgery and immediately snap frozen in liquid nitrogen till further analysis. The details of the clinicopathological features of all OS patients were shown in Table 1.

**Table 1:** Associations between miR-133b/FGFR1 expression and clinicopathological characteristics in patients with osteosarcoma

Clinical parameters	Cases (n)	Expression level		P value	Expression level		P value
		miR-133b^high^	miR-133b^low^	(*P < 0.05)	FGFR1^high^	FGFR1^low^	(*P < 0.05)
Age (years)							
< 18 years	20	13	7	0.0502	8	12	0.0577
≥ 18 years	10	2	8		8	2	
Gender							
Male	15	1	14	< 0.0001	10	5	0.2723
Female	15	14	1		6	9	
Tumor size (cm)							
< 5	12	8	4	0.2635	5	7	0.4572
≥ 5	18	7	11		11	7	
Location							
Femur/Tibia	22	14	8	0.0352	9	13	0.0395
Elsewhere	8	1	7		7	1	
TNM stage							
I	14	12	2	0.0007	4	10	0.0261
II + III	16	3	13		12	4	
Distant metastasis							
Yes	16	4	12	0.0092	13	3	0.0027
No	14	11	3		3	11	

* P < 0.05 was considered significantly different

### Cell culture and treatment

The normal human fetal osteoblast (hFOB 1.19) cells and the human OS cell lines, U2OS, MG-63 and SAOS-2, were purchased from the American Type Culture Collection (Manassas, VA, USA). hFOB 1.19 cells were cultured in DMEM/F12 medium supplemented with 10% fetal bovine serum (FBS) and 0.3 mg/mL G418. The three OS cells lines were cultured in DMEM medium supplemented with 10% and 1% penicillin–streptomycin (all cell culture reagents from Gibco, Carlsbad, CA, USA).

To overexpress miR-133b in OS cells, miR-133b mimics (GenePharma, Shanghai, China) were transfected into target cells using Lipofectamine 2000 (Invitrogen, Carlsbad, CA, USA) following the manufacturer’s instructions. The scrambled miR-133b mimics were used as the negative control (miR-133b NC).

To stably knock down FGFR1 expression, lentiviral transduction particles expressing FGFR1 shRNA (shFGFR1) were purchased from Sigma (St. Louis, MO, USA) and used to infect U2OS or MG-63 cells. Lentiviral transduction particles expressing control shRNA (shNC) were used as the negative control. Stable transfected (shFGFR1 or shNC) cells were established after being selected in 2.5 mg/mL of puromycin (Sigma) for 7 days.

### Reverse transcription-quantitative polymerase chain reaction (RT-qPCR)

Total RNA was extracted from tissues or cells using Trizol reagent (Invitrogen, Carlsbad, CA, USA) according to the manufacturer’s protocols. cDNA was synthesized using SuperScript III reverse transcriptase (Invitrogen) and qPCR performed using PowerUp SYBR Green Master Mix (Applied Biosystems, Foster City, CA, USA) as instructed by the manufacturers. The following primers were used: miR-133b forward primer 5′-AAGAAAGATGCCCCCTGCTC-3′, reverse primer 5′-GTAGCTGGTTGAAGGGGACC-3′; FGFR1 forward primer 5′-AACCTGACCACAGAATTGGAGGCT-3′, reverse primer 5′-ATGCTGCCGTACTCATTCTCCACA-3′; U6 (internal control) forward primer 5′-CTCGCTTCGGCAGCACA-3′, reverse primer 5′-AACGCTTCACGAATTTGCGT-3′. GAPDH (internal control) forward primer 5′-TGTGGGCATCAATGGATTTGG-3′, reverse primer 5′-ACACCATGTATTCCGGGTCAAT-3′. The relative expression of a target gene to that of the internal control was calculated using 2^−ΔΔCt^ method [15].

### Luciferase reporter assay

We used miRanda software (http://www.microrna.org/microrna/getGeneForm.do) and identified a potential binding site to miR-133b within the 3′-UTR of human FGFR1 mRNA. The wild-type (WT) 3′-UTR sequence of FGFR1 mRNA containing the potential miR-133b-binding site and the mutated sequence (MUT) that would disrupt the binding to miR-133b were cloned into pRL-CMV luciferase reporter plasmids separately. Both MG-63 and U2OS cells were co-transfected with the luciferase reporter plasmids (WT or MUT) and miR-133b mimics or control miRNA (miR-133b NC) using Lipofectamine 2000 (Invitrogen) according to the manufacturer’s instructions. At 48 h following the transfection, luciferase activity was detected using the Dual Luciferase Reporter Assay System (Promega, Madison, WI, USA) according to the manufacturer’s instructions.

### 3-(4, 5-dimethylthiazolyl-2)-2, 5-diphenyltetrazolium bromide (MTT) assay

Cells were seeded in 96-well plates at a density of 2 × 10^4^ cells/mL and incubated for 24, 48, or 72 h, respectively. At the end of the incubation, 20 μL of MTT solution (5 mg/mL in PBS, Sigma) was added to each well and the plates were incubated at 37 °C for a further 3 h. The medium was then discarded and 100 µL dimethyl sulfoxide (DMSO, Sigma) was added to each well and incubated for 2 h in the dark at room temperature. DMSO dissolved the formazan crystals and created a purple color. Finally, the optical density (proportional to the number of live cells) was assessed at 570 nm with a Microplate Reader Bio-Rad 550.

### Cell cycle analysis with propidium iodide (PI)

Cells were collected, washed with PBS for three times, and fixed in 70% ethanol overnight. For cell cycle analysis, fixed cells were stained using FxCycle™ PI/RNase Staining Solution (Thermo Fisher Scientific, Waltman, MA, USA) according to the manufacturer’s instructions and analyzed by FACSCalibur (BD Biosciences, San Jose, CA, USA).

### Apoptosis analysis with Annexin-V/PI

Apoptotic cells were detected using Annexin-V-FITC and PI kit (Thermo Fisher Scientific) following the manufacturer’s instructions. The percentage (%) of apoptotic cells was calculated as the  % sum of Annexin-V^+^PI^−^ (representing early apoptotic cells) and Annexin-V^+^PI^+^ cells (representing late apoptotic cells).

### Wound healing migration assay

To perform the migration analysis [16], target cells were plated into 24-well plates and allowed to grow in growth medium to confluence. A 1-mm wide scratch was made across the cell layer using a sterile pipette tip. Plates were photographed immediately (0 h) and at 24 h after scratching at an identical location, respectively, with the width (W) of the scratch measured. The wound closure was calculated as (W_0h_ − W_24h_)/W_0h_ × 100%. All experiments were performed in triplicates for at least three times.

### Transwell migration and invasion assays

To assess cell migration and invasion, Transwell insert (8.0 µm, Corning, Lowell, MA, USA) was either not (for migration assay) or coated with Matrigel (BD Biosciences, San Jose, CA, USA, for invasion assay). The single-cell suspension of target cells was seeded into the top well at 1 × 10^5^ cells/well and cultured in serum-free DMEM medium at 37°C. In the lower chamber, we added 500 μL of DMEM containing 10% FBS as the chemoattractant. After 24 h, the cells remaining on the upper side of the membrane were gently removed with cotton swabs, and the migrated or invaded cells on the lower side of the membrane were fixed in 95% methanol, stained with crystal violet for 5–10 min, and photographed under an inverted microscope (× 100).

### Western blot analysis

Total proteins were extracted from cells using RIPA buffer and separated on SDS-PAGE gel. Following the transfer of separated proteins onto a polyvinylidene difluoride membrane, the membrane was blocked with 5% nonfat milk in TBST (10 mM Tris, pH 8.0, 150 mM NaCl, 0.5% Tween 20) at room temperature for 1 h, washed three times in TBST, and incubated with one of the following primary antibodies (all from Cell Signaling Technology, Danvers, MA, USA, unless otherwise noted) at 4 °C overnight: FGFR1, p-PI3K, PI3K, p-Akt, Akt, Ras, Raf, p-Erk1/2, Erk1/2, N-cadherin, E-cadherin, and GAPDH (internal control). After three washes with TBST, the membrane was incubated with horseradish peroxidase-conjugated secondary antibodies at room temperature for 2 h. The signal was developed using the ECL system (Beyotime, Jiangsu, China) according to the manufacturer’s instructions. The relative expression of a target protein was calculated as the ratio of the signal density of the target protein to that of the internal control.

### Statistical analysis

All data were analyzed by SPSS 13.0 software and presented as mean ± SD. Spearman correlation analysis was performed to analyzed the correlation between miR-133b and FGFR1 in normal or OS tissues. The association between miR-133b/FGFR1 expression and clinicopathological characteristics of OS patients was assessed by the Chi squared test. Statistical evaluation was performed using Student’s *t* test (two-tailed) between two groups or one-way analysis of variance (ANOVA) followed by Tukey post hoc test for multiple comparison. A *P* value of less than 0.05 was considered statistically significant.

## Results

### miR-133b was down-regulated while FGFR1 up-regulated in OS tissues or cell lines

**Fig. 1:**
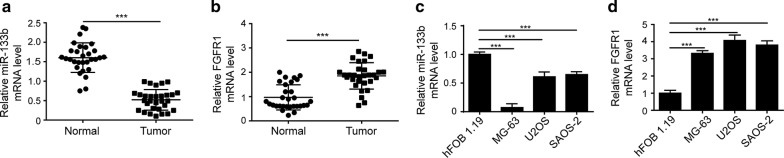
miR-133b was down-regulated while FGFR1 up-regulated in OS tissues or cell lines. The relative mRNA levels of miR-133b (**a**) and FGFR1 (**b**) in 30 pairs of OS tissues and normal tissues were examined by qRT-PCR. **c**, **d** The relative mRNA levels of miR-133b (**c**) and FGFR1 (**d**) in indicated OS cells and normal osteoblasts (hFOB 1.19) were measured by RT-qPCR. ***P < 0.001

Earlier studies reported the down-regulation of miR-133b [10] and the up-regulation of FGFR1 [17] in OS, together with their clinical significance. However, little is known on the crosstalk between miR-133b and FGFR1 in OS. In this study, we first compared the expressions of miR-133b and FGFR1 between 30 OS tissues and paired normal tissues using RT-qPCR. As shown in Fig. 1a, b, miR-133b level was significantly reduced, while FGFR1 level increased in OS tissues, when compared with the paired normal bone tissues. However, the correlation between miR-133b and FGFR1 transcript levels in both OS and normal bone tissues were not statistically significant (data not shown). As shown in Table 1, low miR-133b expression and high FGFR1 expression were associated with location of the malignant lesion (*P *< 0.05), advanced clinical stage (*P *< 0.05), and distant metastasis (*P *< 0.05). Furthermore, we compared the levels of miR-133b and FGFR1 between three well-characterized OS cell lines, MG-63, U2OS, and SAOS-2, and the normal human osteoblast (hFOB 1.19) cells. Consistent with findings from OS tissues, miR-133b was significantly down-regulated, while FGFR1 potently up-regulated in all three OS cells than in normal osteoblast cells (Fig. 1e, f). Taken together, these data suggest that miR-133b and FGFR1 may participate in the OS progression.

### miR-133b directly and essentially controlled FGFR1 expression in OS cells

**Fig. 2:**
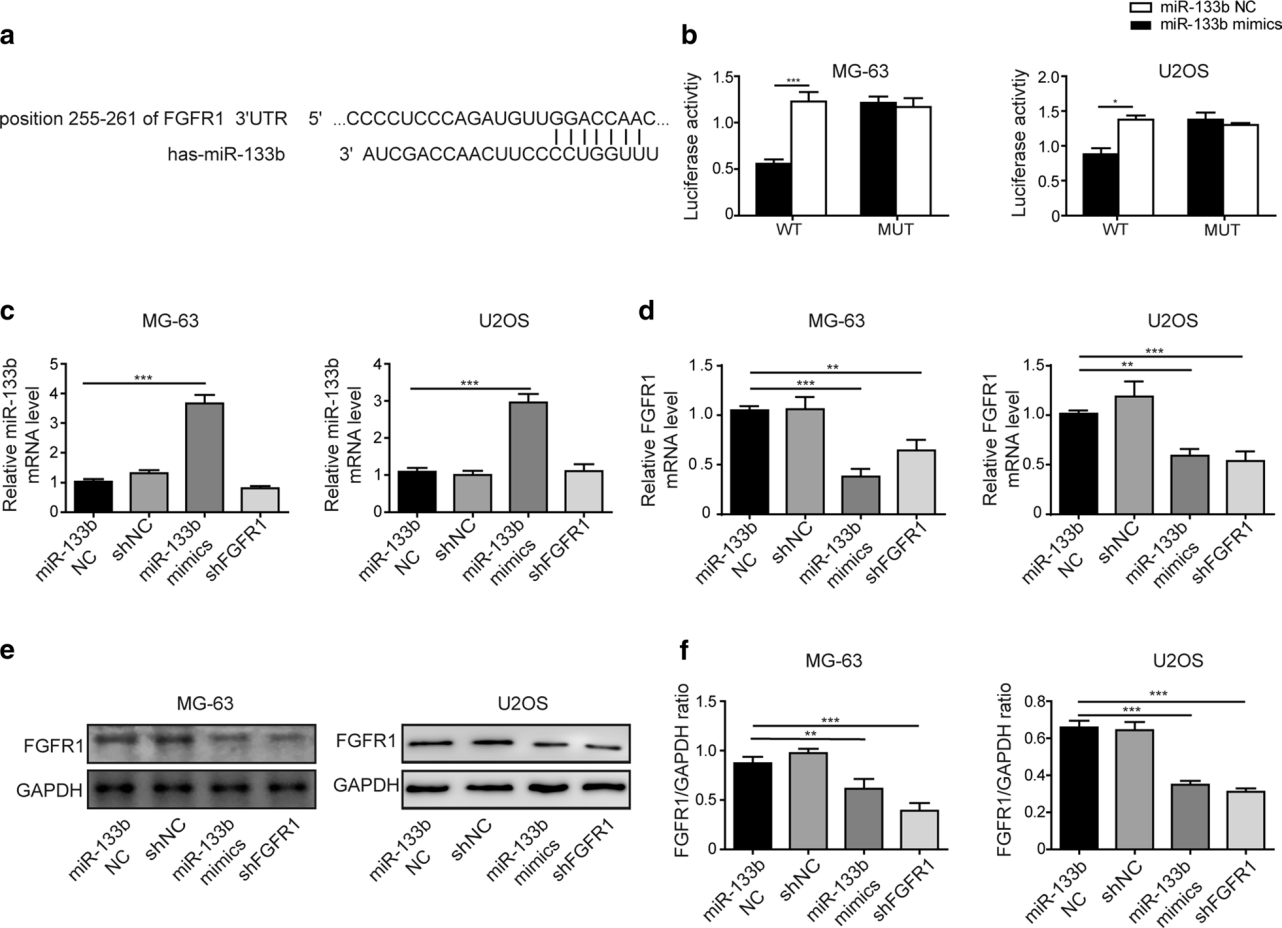
miR-133b directly and essentially controlled FGFR1 expression in OS cells. **a** Bioinformatics predicted the potential binding site between miR-133b and the 3′-UTR of FGFR1. **b** Luciferase assay showed miR-133b directly inhibited FGFR1. The luciferase activities were measured at 48 h after co-transfecting MG-63 or U2OS cells the luciferase reporter driven by either WT or MUT FGFR1 3′-UTR and miR-133b mimics or control miRNA (miR-133b NC). **c**, **d** The relative expressions of miR-133b (**c**) and FGFR1 mRNA (**d**) in MG-63 (left panel) and U2OS cells (right panel) after transfected with miR-133b mimics vs. miR-133b NC, or shFGFR1 vs. shNC were measured by qRT-PCR. **e**, **f** The relative expression of FGFR1 protein in MG-63 (left panel) and U2OS cells (right panel) after transfected with miR-133b mimics vs. miR-133b NC, or shFGFR1 vs. shNC was measured by Western blotting. The representative images of Western blotting were shown in **e** and the quantification of the signals was presented as mean ± standard deviation from three independent experiments shown in **f**. *P < 0.05, **P < 0.01 and ***P < 0.001

A previous study reported that FGFR1 was a direct target gene inhibited by miR-133b in gastric cancer [14]. To examine whether this is also the case in OS cells, we first applied Bioinformatic analysis and identified a potential binding site to miR-133b within the 3′-UTR of human FGFR1 mRNA (Fig. 2a). Next, we generated a mutation within the potential miR-133b-binding site and cloned either the wild-type (WT) or the mutant (MUT) 3′UTR sequence of human FGFR1 upstream of the luciferase reporter gene. As shown in Fig. 2b, miR-133b mimics specifically and potently reduced the luciferase activity driven by WT but not MUT FGFR1 3′UTR sequence in both MG-63 and U2OS cells. To further analyze the mutual regulation between miR-133b and FGFR1, we either overexpressed miR-133b or stably knocked down endogenous FGFR1 with shRNA-mediated gene silencing (shFGFR1) in both U2OS and MG-63 cells. Correspondingly, control miRNA mimics (miR-133b NC) and shRNA (shNC) were used, respectively. We found that miR-133b mimics, in addition to elevating miR-133b level, significantly reduced the endogenous FGFR1 expression to that achieved by shFGFR1, both on the steady-state mRNA (Fig. 2c, d) and the protein levels (Fig. 2e, f). Collectively, these data suggest that miR-133b not only directly binds to the 3′-UTR sequence of human FGFR1 mRNA, but also functionally inhibits its transcription. FGFR1 is a direct target of miR-133b in OS cells.

### miR-133b and FGFR1 essentially regulated the proliferation and apoptosis of OS cells

**Fig. 3:**
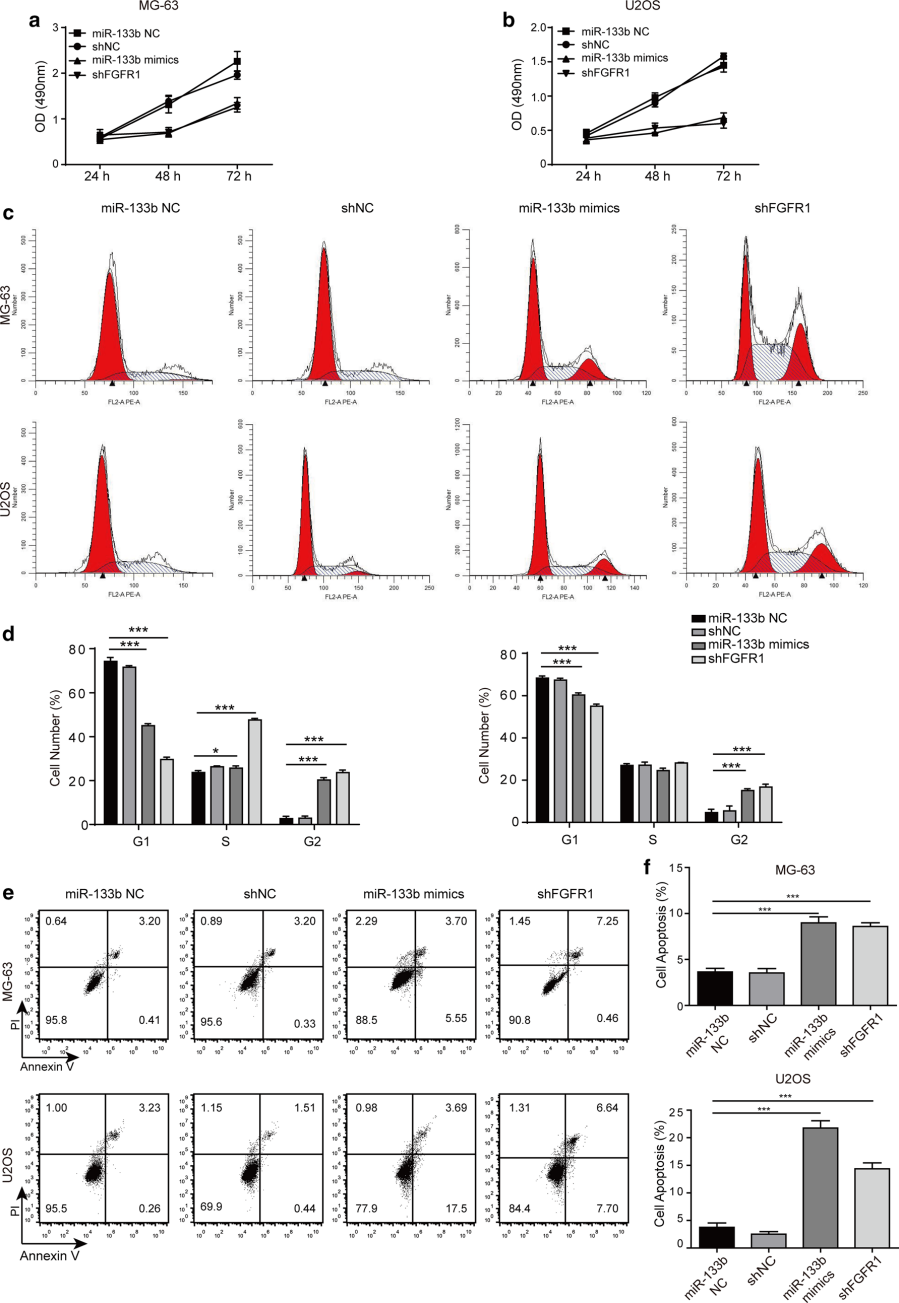
miR-133b and FGFR1 essentially regulated the proliferation and apoptosis of OS cells. The viability of MG-63 (**a**) and U2OS cells (**b**) transfected with miR-133b mimics vs. miR-133b NC, or with shFGFR1 vs. shNC for 24, 48, and 72 h, respectively, was examined by MTT assay. **c** The cell-cycle distribution of MG-63 (upper panels) and U2OS cells (lower panels) transfected with miR-133b mimics vs. miR-133b NC, or with shFGFR1 vs. shNC was determined by flow cytometry analysis. **d** The quantification of the percentage (%) of cells in different phases of cell cycle (G1, S, G2/M) was presented. **e** Apoptosis was examined by staining the indicated cells with Annexin V and PI. **f** The total  % of early apoptotic (Annexin C^+^PI^−^) and late apoptotic (Annexin V^+^PI^+^) cells were quantified from at least three independent experiments. *P < 0.05 and ***P < 0.001

The aberrant expressions of miR-133b and FGFR1 in OS tissues suggest their functional significance during the progression of OS. By monitoring the cell viability by MTT assay, we showed that overexpressing miR-133b or down-regulating FGFR1 was sufficient to reduce cell viability significantly (Fig. 3a, b). Since cell viability results from the balance between cell proliferation and cell death, we continued with assays to determine the effects of overexpressing miR-133b or knocking down FGFR1 on cell-cycle distribution and apoptosis, respectively. As shown in Fig. 3c, d, overexpressing miR-133b or knocking down FGFR1 significantly lowered the number of cells in G1 phase and increased that in G2/M phase, when compared to the corresponding control cells, suggesting an arrest of cell cycle in G2/M phase. In MG-63 cells, shFGFR1 also dramatically increased the number of cells in S phase. Moreover, overexpressing miR-133b or targeting FGFR1 potently stimulated apoptosis, as represented by the percentage of early apoptotic (Annexin V^+^PI^−^) plus late apoptotic (Annexin V^+^PI^+^) cells detected by flow cytometry (Fig. 3e, f). Taken together, these data suggest that both miR-133b and FGFR1 essentially yet oppositely regulate the viability, cell cycle, and apoptosis of OS cells.

### miR-133b and FGFR1 critically controlled the migration and the invasion of OS cells

**Fig. 4:**
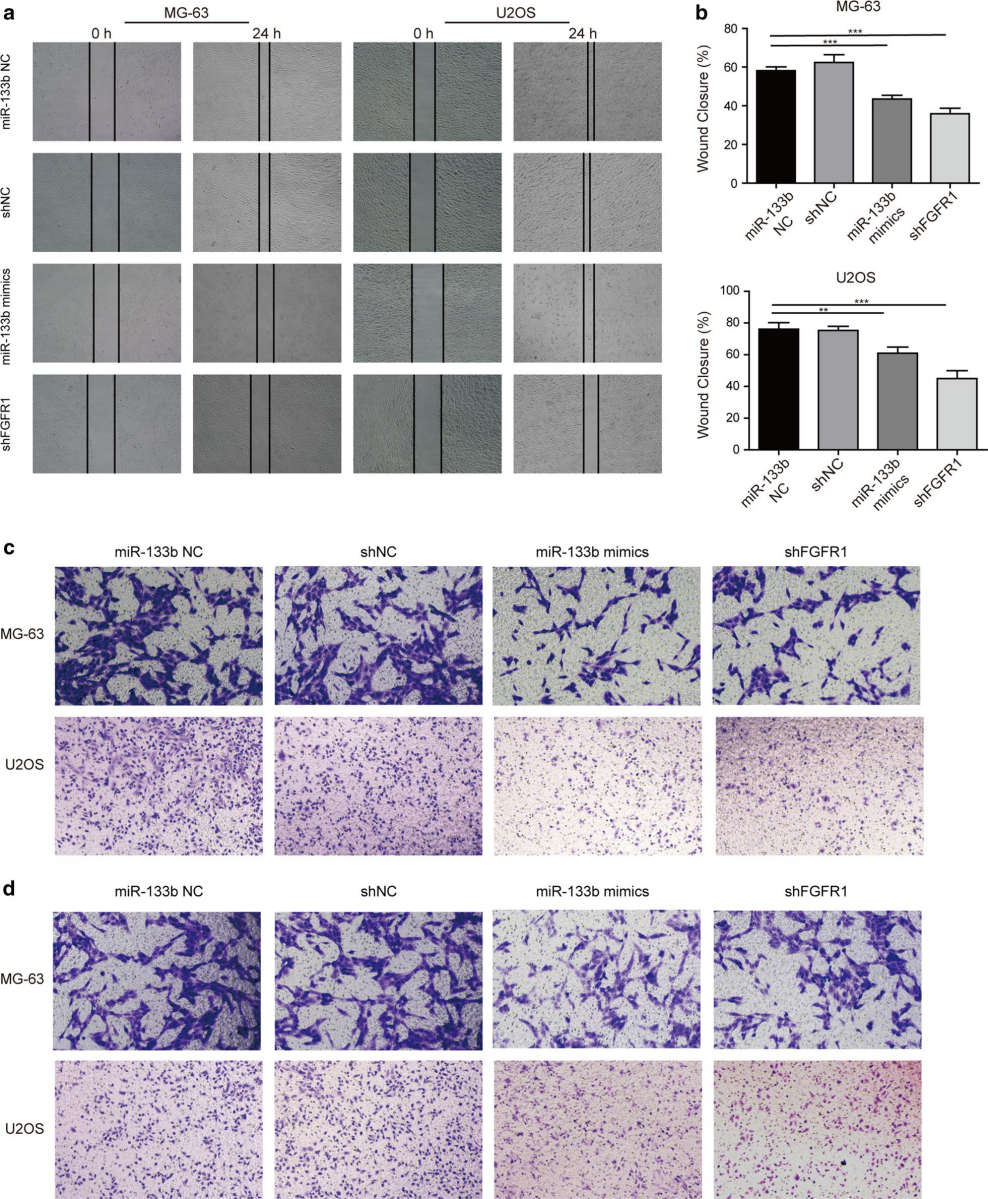
miR-133b and FGFR1 critically controlled the migration and the invasion of OS cells. **a**, **b** The migration of MG-63 and U2OS cells transfected with miR-133b mimics vs. miR-133b NC, or with shFGFR1 vs. shNC was examined by wound healing assay, with representative images shown in **a** and % of wound closure quantified and averaged from three independent experiments shown in **b**. **c**, **d** The representative images of migrated and invaded MG-63 and U2OS cells transfected with miR-133b mimics vs. miR-133b NC, or with shFGFR1 vs. shNC were examined by Transwell migration and invasion assay, respectively. **P < 0.01 and ***P < 0.001

Next, we assessed the effects of overexpressing miR-133b or knocking down FGFR1 on the migration and the invasion of OS cells, two biological processes essential for the aggressive and metastatic growth of OS. Overexpressing miR-133b or knocking down FGFR1 significantly slowed down the closure of wound made on both U2OS and MG-63 cells when compared to the corresponding control cells (Fig. 4a, b). Similarly, either treatment also noticeably inhibited transwell migration (Fig. 4c) and invasion (Fig. 4d) of both OS cells examined. These data suggest that both miR-133b and FGFR1 play essential yet opposing roles in controlling the malignant and metastatic behaviors of OS cells.

### Elevating miR-133b or targeting FGFR1 suppressed Ras/MAPK and PI3K/Akt signaling pathways in OS cells

**Fig. 5:**
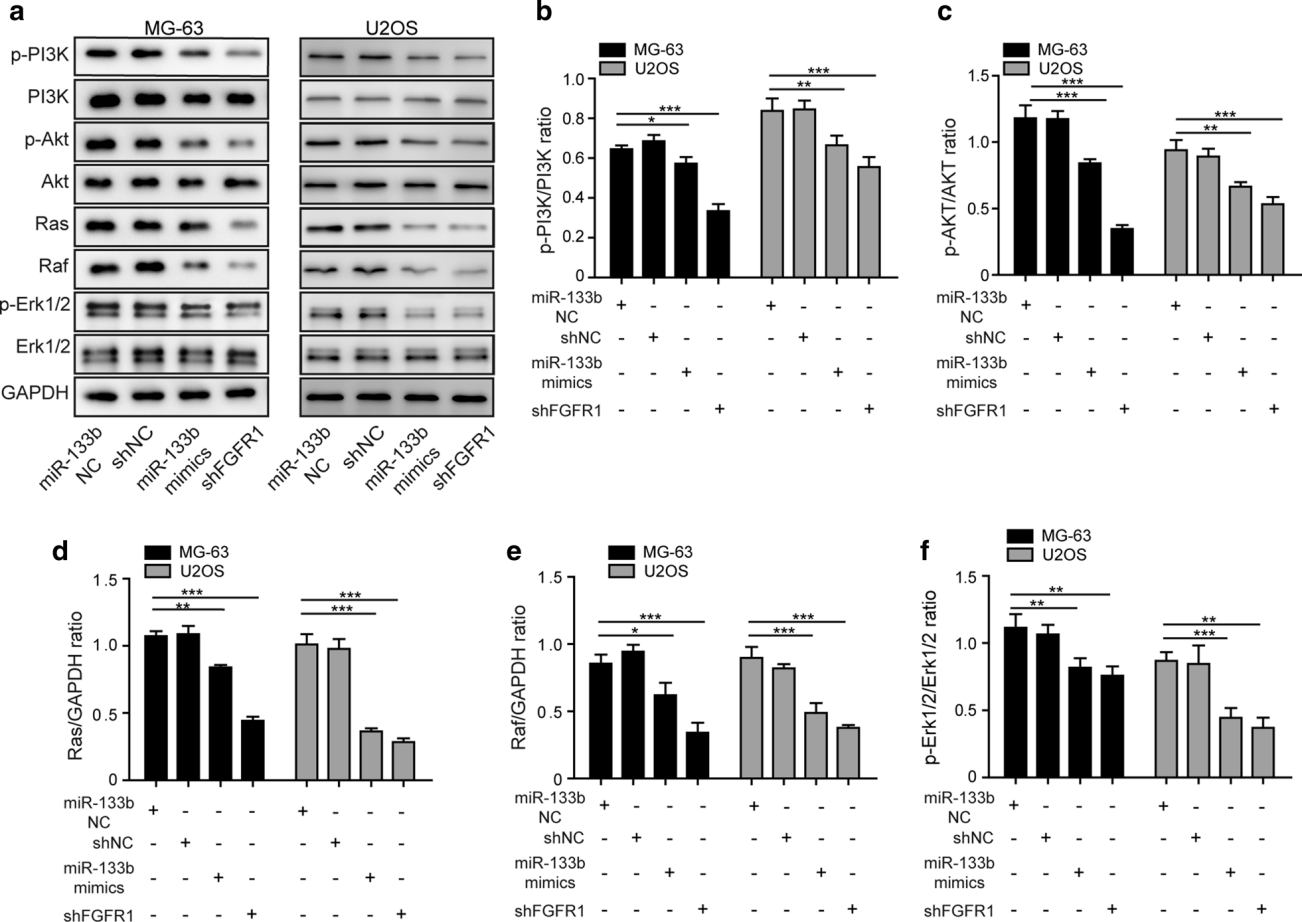
Elevating miR-133b or targeting FGFR1 suppressed Ras/MAPK and PI3K/Akt signaling pathways in OS cells. **a** The levels of FGFR1, p-PI3K, PI3K, p-Akt, Akt, Ras, Raf, p-Erk1/2, and Erk1/2 in MG-63 or U2OS cells transfected with miR-133b mimics vs. miR-133b NC, or with shFGFR1 vs. shNC were examined by Western blotting. **b**–**f** The quantification of p-PI3K, p-Akt, Ras, Raf, and p-Erk1/2 from indicated samples, respectively. *P < 0.05, **P < 0.01 and ***P < 0.001

It is well known that deregulated FGF/FGFR activations are closely associated with cancer progression [12]. The oncogenic effects of FGFRs are mediated by activating multiple downstream signal transduction pathways, including Ras/MAPK and PI3K/Akt [11, 12]. To explore the signaling pathways controlled by miR-133b/FGFR1 in OS cells, we examined the activation/phosphorylation status of key signaling molecules of these pathways. As shown in Fig. 5a–f, in both MG-63 and U2OS cells, overexpressing miR-133b or knocking down FGFR1 reduced the phosphorylation of PI3K, Akt, and Erk1/2, and decreased Ras and Raf levels, suggesting that miR-133, by targeting FGFR1, inhibits the activation of Ras/MAPK and PI3K/Akt signaling pathways in OS cells.

### Overexpressing miR-133b or knocking down FGFR1 suppressed EMT of OS cells

**Fig. 6:**
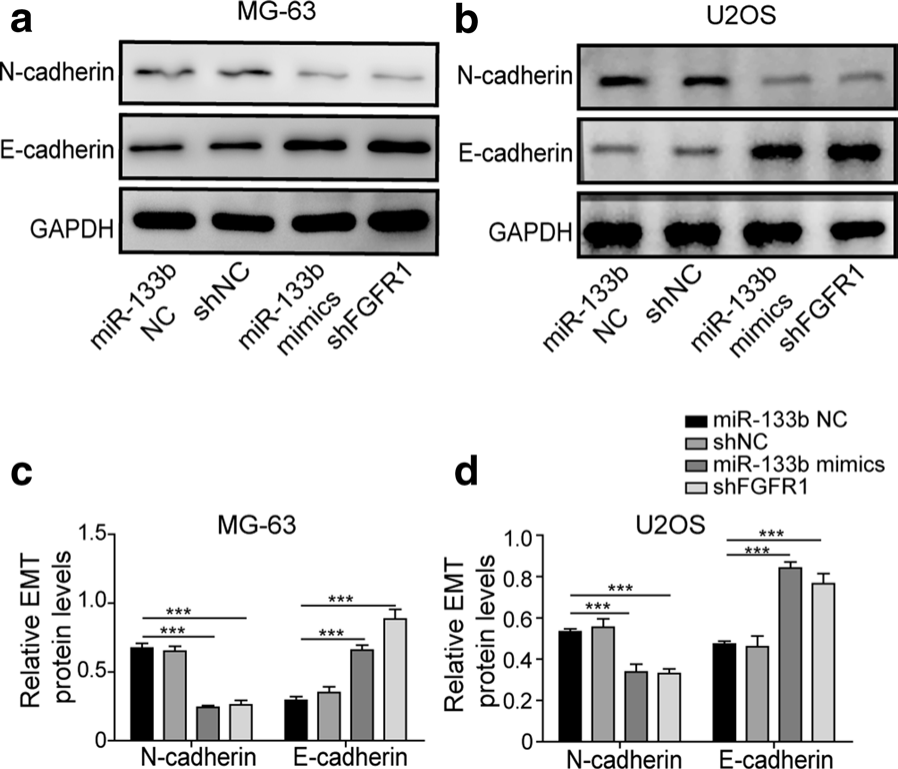
Overexpressing miR-133b or knocking down FGFR1 suppressed EMT of OS cells. MG-63 (**a** and **c**) or U2OS (**b** and **d**) cells were transfected with miR-133b mimics vs. miR-133b NC, or with shFGFR1 vs. shNC. The expressions of N-cadherin and E-cadherin were examined by Western blotting. Representative Western images were shown in **a** and **b** and the quantification of N-cadherin and E-cadherin levels from three independent experiments in **c** and **d**, respectively. ***P < 0.001

The effects of miR-133b/FGFR1 on the viability, the migration, and the invasion of OS cells promoted us to examine their roles on EMT, a biological process whereby epithelial cancer cells acquire mesenchymal phenotypes and initiate the invasive/metastatic cascades [18]. Upon overexpressing miR-133b or knocking down FGFR1 in both MG-63 and U2OS cells, we observed significant down-regulation of N-cadherin (a mesenchymal biomarker) and the up-regulation of E-cadherin (an epithelial marker) in both OS cells (Fig. 6), indicating the suppression of EMT.

## Discussion

OS is an aggressive bone malignancy associated with a high incidence of pulmonary metastasis and poor prognosis, and in most cases threatens the life of children and adolescents [19]. In this study, we confirmed the previous finding that miR-133b was a biologically significant tumor suppressor miRNA in OS [10]. Furthermore, we showed for the first time that FGFR1 was a direct miR-133b target in OS cells, and the miR-133b/FGFR1 axis essentially regulated multiple malignant behaviors of OS cells, including viability, proliferation, apoptosis, migration/invasion, and EMT. We also demonstrated that the Ras/MAPK and PI3K/Akt pathways were controlled by miR-133b/FGFR1 axis in OS cells.

miR-133b has been identified as a tumor suppressor miRNA in various cancers such as gastric cancer [14, 20], lung cancer [21, 22], bladder cancer [23], prostate cancer [24, 25], ovarian cancer [26], and colorectal cancer [27–30]. In OS, Novello et al., by profiling miRNAs between normal and OS tissues, and also between low-grade and high-grade OS tissues, identified 12 differentially expressed miRNAs. Among them, miR-133b was significantly down-regulated in samples with higher malignancy than in those with lower malignancy or in normal samples [31]. Further analysis in U2OS cells showed that miR-133b was sufficient to block cell cycle at G1 phase and induce apoptosis [31]. Another study by Zhao et al. revealed 43 differentially expressed miRNAs between human OS tissues and normal skeletal muscles, among which, miR-133a and miR-133b were robustly down-regulated in OS tissues [10]. Consistent with these findings, here we showed that miR-133b expression was significantly reduced in OS tissues, when compared to matching tumor-free bone tissues, and also in three different OS cells lines when compared to normal osteoblasts. Functionally, using U2OS and MG-63 as the model system, we found that overexpressing miR-133b significantly reduced cell viability, arrested cell cycle progression at G2 phase, and promoted apoptosis. More importantly, over-expressing miR-133b suppressed EMT of OS cells, as represented by inhibited migration/invasion, up-regulated E-cadherin, and down-regulated N-cadherin expression. EMT is a reversible yet important biological process critically contributing to cancer progression, metastasis, and/or drug resistance in cancers [32]. Although OS cells are derived from mesenchymal cells and thus equipped with some mesenchymal features, the presence of EMT and its correlation with aggressive behaviors of OS supports that OS cells are in a metastable status and EMT empowers these cells with higher migratory and invasive capacities [33]. Corresponding to the biological significance of miR-133b in OS, aberrant expression of miR-133b became a potential prognostic marker of human OS [34]. Taken together, these data demonstrated that miR-133b was a pleiotropic tumor suppressor miRNA in OS. In contrast to suppressing tumorigenesis- and metastasis-related biological behaviors, a recent study showed that miR-133b level was up-regulated in cisplatin-resistant OS cells, and miR-133b inhibited apoptosis and promoted migration/invasion under cisplatin stress and thus induced chemoresistance of OS cells [35]. It would be interesting to identify the molecular switch that changes miR-133b from a tumor suppressor miRNA in cisplatin-sensitive OS cells to a chemoresistance-stimulating miRNA in cisplatin-resistant OS cells.

A variety of direct target genes have been identified for carrying out different anti-cancer activities of miR-133b in human cancers. miR-133b regulated cell proliferation and apoptosis by targeting members in tumor necrosis factor-related apoptosis-inducing ligand (TRAIL) pathways such as death receptor 5, Fas apoptosis inhibitory molecule (FAIM), and antiapoptotic enzyme detoxifying protein glutathione-S-transferase pi (GSTP1) [36, 37], or Bcl-2 family members such as Bcl-2, MCL-1, Bcl-wL, and Bcl-xL [38, 39]. It altered energy metabolism of cancer cells by targeting pyruvate kinase isoform M2 (PKM2) [40]. It inhibited migration/invasion by targeting matrix metalloproteinases (MMPs), Fascin actin-bundling protein 1 (FSCN1), or C-X-C motif chemokine receptor 4 (CXCR4) [29, 41, 42]. In this study, we showed that FGFR1 was a direct target gene inhibited by miR-133b in OS cells. More importantly, low miR-133b expression and high FGFR1 expression were associated with location of the malignant lesion, advanced clinical stage, and distant metastasis. Functionally, overexpressing miR-133b or down-regulating FGFR1 presented the same phenotypes, reducing cell viability, arresting cell proliferation, promoting apoptosis, suppressing migration/invasion, and blocking EMT. Extensive studies on FGFR1 have well documented its oncogenic features in different types of human cancer, including blocking apoptosis and promoting stemness, proliferation, drug resistance, migration/invasion, EMT, and angiogenesis [13]. Therefore, data from this study strongly support that in OS cells, inhibiting FGFR1 empowers miR-133b with a plethora of anti-tumorigenic and anti-metastatic activities. Wen et al. presented similar findings in gastric cancer, where they showed that the level of miR-133b negatively correlated with that of FGFR1 in gastric cancer tissues, miR-133b directly inhibited FGFR1 on the protein but not the mRNA level, and by targeting FGFR1, miR-133b inhibited cancer cell growth [14]. In this study, we found that overexpressing miR-133b significantly reduced both FGFR1 mRNA and protein, suggesting that in OS cells, miR-133b targeted FGFR1 expression by mRNA degradation as well as by translational silencing. It also implied that the silencing mechanisms by which miR-133b acts on a particular target gene may vary with cancer-specific microenvironment.

The amplification of multiple FGFR members, including FGFR1, FGFR2, and FGFR3, have been reported in OS [43]. Of these members, FGFR1 amplification was specific for OS patients showing poor responses, but not for those responding well to chemotherapy. FGFR3 level was also associated with poor prognosis [12]. Upon binding to and being activated by different FGFs, FGFRs signal the diversity of functional phenotypes through four major intracellular signaling pathways: Ras/MAPK, PI3K/Akt, PLCγ, and STAT [11, 13]. In this study, we showed that not only down-regulating FGFR1 but also overexpressing miR-133b that acted preferentially through inhibiting FGFR1, were sufficient to suppress the activation of both Ras/MAPK and PI3K/Akt pathways. Although it is still not clear how inhibiting Ras/MAPK and PI3K/Akt pathways result in miR-133b-induced phenotypes, these data suggest that in OS cells, FGFR1 plays an essential role in activating Ras/MAPK and PI3K/Akt pathways. It would be interesting to identify miRNAs or other silencing mechanisms for FGFR2 and/or FGFR3 and explore the therapeutic potential of simultaneously targeting multiple FGFRs in OS cells.

In conclusion, we demonstrated that miR-133b was a pleiotropic tumor suppressor miRNA and targeting FGFR1 as well as the subsequent Ras/MAPK and PI3K/Akt pathways critically mediated the anti-cancer activities of miR-133b in OS cells, including suppressing cell viability, proliferation, migration/invasion, and EMT, and promoting apoptosis. Therefore, both miR-133b and FGFR1 are potential therapeutic targets for OS. Future studies may assess the therapeutic potential of boosting miR-133b expression or inhibiting FGFR1 expression in a preclinical animal OS model. Specifically, focus should be directed toward the therapeutic benefits of altering miR-133b/FGFR1 axis on metastatic behaviors of OS, since metastasis is responsible for the minimal survival and the poor prognosis associated with OS. Lastly, further mechanistic studies into the downstream signaling cascades of miR-133b/FGFR1 axis will help to fine-tune the biological effects of this axis and thus generate therapies with higher specificity and lower side effects.
